# An Assessment of Fixed and Native Chromatin Preparation Methods to Study Histone Post-Translational Modifications at a Whole Genome Scale in Skeletal Muscle Tissue

**DOI:** 10.1186/s12575-017-0059-0

**Published:** 2017-08-29

**Authors:** Sarah-Anne David, Benoît Piégu, Christelle Hennequet-Antier, Maëlle Pannetier, Tiphaine Aguirre-Lavin, Sabine Crochet, Thierry Bordeau, Nathalie Couroussé, Aurélien Brionne, Yves Bigot, Anne Collin, Vincent Coustham

**Affiliations:** 1 URA, INRA, 37380 Nouzilly, France; 2 UMR PRC, CNRS, IFCE, INRA, Université de Tours, 37380 Nouzilly, France; 3 UMR BDR, INRA, ENVA, Université Paris-Saclay, 78350 Jouy-en-Josas, France

**Keywords:** Epigenetics, Histone post-translational modifications, Chromatin immunoprecipitation, Cross-linking, Native, Skeletal muscle

## Abstract

**Background:**

Genomic loci associated with histone marks are typically analyzed by immunoprecipitation of the chromatin followed by quantitative-PCR (ChIP-qPCR) or high throughput sequencing (ChIP-seq). Chromatin can be either cross-linked (X-ChIP) or used in the native state (N-ChIP). Cross-linking of DNA and proteins helps stabilizing their interactions before analysis. Despite X-ChIP is the most commonly used method, muscle tissue fixation is known to be relatively inefficient. Moreover, no protocol described a simple and reliable preparation of skeletal muscle chromatin of sufficient quality for subsequent high-throughput sequencing. Here we aimed to set-up and compare both chromatin preparation methods for a genome-wide analysis of H3K27me3, a broad-peak histone mark, using chicken *P. major* muscle tissue.

**Results:**

Fixed and unfixed chromatin were prepared from chicken muscle tissues (*Pectoralis major*). Chromatin fixation, shearing by sonication or digestion and immunoprecipitation performed equivalently. High-quality Illumina reads were obtained (q30 > 93%). The bioinformatic analysis of the data was performed using epic, a tool based on SICER, and MACS2. Forty millions of reads were analyzed for both X-ChIP-seq and N-ChIP-seq experiments. Surprisingly, H3K27me3 X-ChIP-seq analysis led to the identification of only 2000 enriched regions compared to about 15,000 regions identified in the case of N-ChIP-seq. N-ChIP-seq peaks were more consistent between replicates compared to X-ChIP-seq. Higher N-ChIP-seq enrichments were confirmed by ChIP-qPCR at the *PAX5* and *SOX2* loci known to be enriched for H3K27me3 in myotubes and at the loci of common regions of enrichment identified in this study.

**Conclusions:**

Our findings suggest that the preparation of muscle chromatin for ChIP-seq in cross-linked conditions can compromise the systematic analysis of broad histone marks. Therefore, native chromatin preparation should be preferred to cross-linking when a ChIP experiment has to be performed on skeletal muscle tissue, particularly when a broad source signal is considered.

**Electronic supplementary material:**

The online version of this article (doi:10.1186/s12575-017-0059-0) contains supplementary material, which is available to authorized users.

## Background

Histone post-translational modifications (HPTM) such as methylation and acetylation of lysine residues are widely studied epigenetic marks. The genomic distribution of histone marks is commonly analyzed by immunoprecipitation (IP) of the chromatin using HPTM-specific antibodies, followed by quantitative PCR (ChIP-qPCR) or high throughput sequencing (ChIP-seq). Two main methods exist to perform IP either based on cross-linking (X-ChIP) or native (N-ChIP) chromatin preparation. X-ChIP is the most common technique and consists in a covalent fixation of the interactions between proteins and DNA using cross-linking reagents such as formaldehyde. N-ChIP is based on unfixed chromatin and therefore requires stable interactions between DNA and proteins such as histones [1].

HPTM were shown to affect gene expression by altering the chromatin accessibility to the transcriptional machinery [2]. Recent advances in genome-wide sequencing technologies enabled systematic mapping of histone marks in a large range of tissues leading to an improved understanding of the interplay between histone marks and their functions [3]. However, whole genome analysis of epigenetic modifications can still be challenging due to their various distribution on the genome. HPTM signals are mostly categorized to either narrow peaks (highly localized signals, such as H3K4me3) or broad peaks (spanning large genomic domains, such as H3K27me3 or H3K36me3) [4, 5]. Broad peak marks such as the trimethylation of the lysine 27 on the histone H3 (H3K27me3) tend to display flatter cross-correlation profiles than narrow peaks that complicates their analysis [6]. Therefore, it was recommended to maximize site discovery by optimizing IP and sequencing deeply, within reasonable expense constraint [6].

Skeletal muscles are complex heterogeneous tissues formed by the association of several types of fibers and to a lesser extent of undifferentiated satellite cells. Muscle fibers are poly-nucleated cells and their nuclei are embedded in actin/myosin filaments that ensure the contractility of this organ for voluntary movement and skeleton support [7]. Due to its nature, cross-linking of skeletal muscle tissue was reported to be relatively inefficient compared to most other tissues [8]. It was suggested that the myofiber structure may act as a physical barrier that limits the access of the cross-linking reagent to the nuclei. A muscle relaxation treatment right after sampling was recently proposed to improve nuclei accessibility to the fixation reagent [9, 10]. Another study reported an enzymatic digestion method to separate mature myofibers from satellite cells allowing simultaneous preparation of chromatin of nuclei from both cell types [8]. However, this labor-intensive protocol required several steps before fixation including the digestion and separation of the cells that may ultimately alter chromatin integrity. N-ChIP on the contrary could overcome the issue of crosslinking incompatibilities of the muscle tissue as it is based on native, unfixed, chromatin. However N-ChIP is not suitable to study proteins that are not stably bound to the DNA such as transcriptional factors, and is therefore less preferred in integrative studies [11–13]. Nonetheless, a few studies have successfully reported ChIP-seq profiles of HPTM from muscle tissues. X-ChIP-seq was performed on muscles to study the narrow peaks marks H3K4me3 and H3ac in rats [14] and H3K27ac in mice [15]. N-ChIP-seq was used rather than X-ChIP-seq to study the broad source mark H3K27me3 on bovine muscle [16].

Given the absence of a standardized ChIP-seq protocol for skeletal muscle tissue to study histone marks, we sought to compare two chromatin preparation strategies based either on cross-linked or native chromatin preparation. In agreement with previous studies and ENCODE recommendations we used relaxation buffer for X-ChIP to improve chromatin fixation and performed several optimization steps throughout the protocols [6, 9, 10]. We explored the impact of both chromatin preparations on the genome-wide distribution of H3K27me3 through Illumina sequencing. Our analysis suggested that N-ChIP-seq is more efficient to discover H3K27me3 regions of enrichment than X-ChIP-seq for a given number of reads when performed on chicken skeletal muscle.

## Methods

### Animals

Cobb 500 male chickens were raised in the INRA UE1295 PEAT experimental facilities (Pôle d’Expérimentation Animale de Tours, Agreement N° C37–175-1). Experiments were performed in accordance with the legislation governing the ethical treatment of birds and were approved by the French Ministry of Higher Education and the Val-de-Loire Animal Ethics Committee (Authorization N° APAFIS#4608–201603211212171 v2). *Pectoralis Major* muscles were sampled at 35 days of age after slaughter from male chickens for ChIP-seq experiments. Two animals were used for muscle X-ChIP-seq and two for muscle N-ChIP-seq. For ChIP-qPCR experiments, *Pectoralis Major* muscles of three male animals of the same age were sampled: one half of each sample was fixed for X-ChIP-qPCR and the other half was left untreated for N-ChIP-qPCR.

### Cross-Linking Chromatin Immunoprecipitation

#### Chromatin Preparation

Muscles were chopped in 250 mg pieces at sampling and were subsequently incubated in RBI buffer (10 mM KCl, 5 mM MgCl2, 5 mM EGTA pH 8, 5 mM Na pyrophosphate, 1 mM PMSF, 1 X Complete™ protease inhibitors from Roche Diagnostics, Basel, Switzerland; [9]) for 30 min on ice. The preparation of chromatin was adapted from Coustham et al. [17]. Samples were centrifuged 5 min at 3000 *g* and pellets were resuspended in ice-cold Phosphate Buffered Saline (PBS, Sigma-Aldrich, Saint-Louis, USA) with 1 X Complete™ protease inhibitors (PBS-C) containing 1 or 2% formaldehyde (FA, Sigma-Aldrich, Saint-Louis, USA) for 5, 15 or 30 min at room temperature under agitation. Reactions were stopped by adding glycine to a final concentration of 125 mM for 5 min at room temperature under agitation. Tubes were centrifuged 5 min at 3000 *g* and the pellet was washed 3 times in ice-cold PBS-C. At this step samples can be stored in PBS-C 30% glycerol and washed in cold PBS-C after thawing. Samples were ground using an ice-cold mortar and pestle until preparations became homogeneous and incubated 10 min on ice with 1 mL of ice-cold Lysis Buffer (10 mM Tris-HCl pH 8, 5 mM EDTA pH 8, 85 mM KCl, 0.5% NP-40, 1 mM PMSF, 1 X Complete™ protease inhibitors). Tissues were further homogenized using a dounce homogenizer 20 times and divided into four 250 μL fractions in 1.5 mL TPX tubes (Diagenode, Denville, USA). Cells were centrifuged 5 min at 5000 *g* and cellular pellets were resuspended in 300 μL sodium Dodecyl Sulphate (SDS) Lysis Buffer (50 mM Tris-HCl pH 8, 10 mM EDTA pH 8, 1% SDS, 10% glycerol, 1 X complete™ protease inhibitors). Tubes were vortexed 20 s, incubated 10 min on ice and vortexed 20 s again. Lysed nuclei were sonicated using a Bioruptor (Diagenode, Denville, USA) set to high setting (30 s ON, 30 s OFF; 2 × 5 min to 6 × 5 min). Samples were then centrifuged 5 min at 10600 *g* and stored at −80 °C. Optimizations of fixation and sonication parameters are shown in Additional file 1: Fig. S1a.

#### Chromatin Quantification, Reverse Cross-Linking and Quality Assessment

Muscle chromatin concentrations were estimated both by Qubit Fluorometric Quantitation (Qubit dsDNA Assay Kit, Thermo Scientific, Waltham, USA) and by spectrophotometry using a NanoDrop ND-1000 Spectrophotometer (Thermo Scientific, Waltham, USA). Cross-linking was reversed before gel migration of the chromatin. To that end 10 μL of supernatant was diluted 4 times in UltraPure water and NaCl was added to the final concentration of 200 mM. Samples were incubated overnight in a Thermomixer comfort (Eppendorf, Hamburg, Germany; 65 °C, 1400 rpm) then incubated with RNase A (Thermo Scientific, Waltham, USA) 10 min at 45 °C under agitation (1400 rpm) followed by a proteinase K incubation (Qiagen, Hilden, Germany; 1 h at 45 °C, 1400 rpm). DNA purification was performed using a Macherey Nagel NucleoSpin® Gel and PCR Clean-up kit (Macherey Nagel, Duren, Germany) following supplier’s protocol. DNA smears were visualised by migration on a 1% agarose gel electrophoresis stained with Gel Red (Biotium, Fremont, USA).

#### Immunoprecipitation

Immunoprecipitation protocol was adapted from Coustham et al. [17]. LoBind tubes (Eppendorf, Hamburg, Germany) were used throughout the IP. For each sample, 10 μL of dynabeads-protein A (Invitrogen, Carlsbad, USA) were washed twice on a magnetic stand (Invitrogen, Carlsbad, USA) with 1 mL of ChIP dilution buffer (CDB; 1.1% Triton X-100, 1.2 mM EDTA pH 8, 16.7 mM Tris-HCl pH 8, 167 mM NaCl, 1 X Complete™ protease inhibitors). Five microliters of anti-H3K27me3 antibody (07–449, lot #2506493 Merck-Millipore, Billerica, USA) or 5 μL of CDB (no-antibody control) were added to 45 μL of CDB and were incubated for 2 h on a rotating wheel at 4 °C. The beads were quickly spun using a table-top centrifuge (6 K), placed on a magnetic stand and the supernatant was discarded. The beads were washed 3 times with 1 mL of CDB and incubated 5 min on a rotating wheel at 4 °C between each wash. The beads were resuspended in 50 μL of CDB. For each IP, 100 μL of chromatin was added to 850 μL of CDB and 50 μL of mix CDB plus beads. 30 μL of chromatin was stored at −80 °C for the input fraction. Samples were homogenized and incubated overnight on a rotating wheel at 4 °C. Samples were washed twice using 1 mL of ice-cold Low Salt Wash Buffer (150 mM NaCl, 0.1% SDS, 1% Triton X-100, 2 mM EDTA pH 8, 20 mM Tris-HCl pH 8), once using 1 mL of ice-cold High Salt Wash Buffer (500 mM NaCl, 0.1% SDS, 1% Triton X-100, 2 mM EDTA pH 8, 20 mM Tris-HCl pH 8), once using 1 mL of ice-cold Lithium Chloride Wash Buffer (250 mM LiCl, 1% NP-40, 1% sodium deoxycholate, 1 mM EDTA pH 8, 10 mM Tris-HCl pH 8) and finally once using 1 mL of ice-cold TE Buffer (10 mM Tris-HCl pH 8, 1 mM EDTA pH 8). Beads were transferred into a new tube to reduce background noise and washed once in 1 mL of ice-cold TE Buffer for 5 min on a rotating wheel at 4 °C. Reverse cross-linking of samples were realised as described above in a final volume of 240 μL. DNA purification was performed using a Macherey Nagel NucleoSpin® Gel and PCR Clean-up kit (Macherey Nagel, Duren, Germany) following supplier’s protocol (two successive elution of 20 μL each were realised in NE buffer and pooled).

### Native Chromatin Immunoprecipitation

**The protocol used for the preparation of the chromatin and the IP was adapted from Wagschal et al.** [18].

#### Chromatin Preparation

Approximately 800 mg of *Pectoralis Major* muscle samples were ground in liquid nitrogen using an A11 basic grinder (IKA, Staufen im Breisgau, Germany). Tissues were further homogenized using a dounce homogenizer in 7 mL of nuclei preparation buffer 1 (60 mM KCl, 15 mM NaCl, 5 mM MgCl2, 0.1 mM EGTA, 15 mM Tris-HCl pH 7.5, 0.3 M sucrose, 0.5 mM DTT, 0.1 mM PMSF, 3.6 ng/mL aprotinin, 5 mM sodium-butyrate). Samples were then filtered through two layers of sterile muslin cheese cloth, moistened beforehand with buffer 1, in 15 mL tubes (Corning, USA) and centrifuged 10 min at 5432 *g* at 4 °C. The pellet was resuspended in 3 mL of ice-cold nuclei preparation buffer 1. One mL of ice-cold nuclei preparation buffer 2 (buffer 1, 0.8% NP-40) was added. Two equal mixes of 2 mL were gently transferred into two tubes of 13 mL Cultubes (Simport, Beloeil, Canada) containing 8 mL of ice-cold nuclei preparation buffer 3 (60 mM KCl, 15 mM NaCl, 5 mM MgCl2, 0.1 mM EGTA, 15 mM Tris-HCl pH 7.5, 1.2 M sucrose, 0.5 mM DTT, 0.1 mM PMSF, 3.6 ng/mL aprotinin, 5 mM sodium-butyrate), incubated 6 min on ice and centrifuged 20 min at 9289 *g* at 4 °C. The supernatant was carefully removed and the pellet was resuspended in ice-cold MNase digestion buffer (0.32 M sucrose, 50 mM Tris-HCl pH 7.5, 4 mM MgCl2, 1 mM CaCl2, 0.1 mM PMSF, 5 mM sodium-butyrate) to a final volume of 1 mL. The chromatin concentration was estimated using a NanoDrop ND-1000 in 0.1% SDS. Samples were split in fractions containing 100 μg of chromatin in 500 μL of MNase digestion buffer (at this step samples can be stored at −80 °C). The Nuclease S7 Micrococcal nuclease (MNase, Roche Diagnostics, Basel, Switzerland) was added to each sample and incubated at 37 °C (10 U - 7 min or 10 U - 10 min or 20 U - 10 min or 10 U - 15 min or 20 U - 15 min, Additional file 1: Fig. S1b). EDTA (18 mM final) was added to stop the reaction. Samples were centrifuged 10 min at 18516 *g* at 4 °C and the supernatant containing soluble chromatin fragments was retrieved. Chromatin concentration was estimated using a NanoDrop ND-1000. MNase digestion efficiency was verified by migration of 10 μL of supernatant on a 1% agarose gel electrophoresis in 1X DNA Gel Loading Dye supplemented with 6% SDS.

#### Chromatin Immunoprecipitation

IPs were performed on 5 μg of chromatin in 1 mL of ChIP buffer (20 mM Tris-HCl pH 7.5, 20 mM sodium-butyrate, 5 mM EDTA, 0.1 mM PMSF, 50 mM NaCl). LoBind tubes were used throughout the IP. A total of 15 μg of fragmented chromatin was diluted in 3 mL of ChIP buffer and split in 3 equal fractions corresponding to no antibody, H3K27me3 and input fractions. The input fraction was stored at 4 °C until DNA purification. Five μL of anti-H3K27me3 antibody were added to the H3K27me3 fraction (07–449, lot #2506493, Merck-Millipore, Billerica, USA). The H3K27me3 and the no-antibody control fractions were both incubated overnight on a rotating wheel (4 °C). After adding 50 μL of dynabeads-protein A (Invitrogen, Carlsbad, USA), the samples were incubated 4 h on a rotating wheel at 4 °C and then washed with buffers containing increasing concentration of NaCl. Five consecutives washes of 1 mL using washing buffer A (50 mM Tris-HCl pH 7.5, 10 mM EDTA, 5 mM sodium-butyrate, 75 mM NaCl), buffer B (50 mM Tris-HCl pH 7.5, 10 mM EDTA, 5 mM sodium-butyrate, 125 mM NaCl) and buffer C (50 mM Tris-HCl pH 7.5, 10 mM EDTA, 5 mM sodium-butyrate, 175 mM NaCl) were performed using a magnetic stand. Elution was done in 500 μL of elution buffer (50 mM NaCl, 20 mM Tris-HCl pH 7.5, 20 mM sodium-butyrate, 5 mM EDTA, 0.1 mM PMSF, 1% SDS). Half of the input fraction (500 μL corresponding to 2.5 μg of chromatin) was supplemented with SDS to 1% final concentration (*v*/v). All the fractions were then incubated 30 min at room temperature on a rotating wheel. LoBind tubes were then centrifuged 3 min at 1122 *g* and the supernatant containing the DNA was retrieved into a Phase Lock Gel (5 PRIME, Hamburg, Germany) tube. DNA was purified by adding 1 volume of phenol:chloroform:isoamyl alcohol (25:24:1, v:v:v, Sigma-Aldrich, Saint-Louis, USA). Tubes were centrifuged 15 min at 18516 *g*. DNA was precipitated by adding 25 μL of NaCl 5 M, 1 volume of isopropylic alcohol (Sigma-Aldrich, Saint-Louis, USA) and 1 μL of 20 mg/mL glycogen (Invitrogen, Carlsbad, USA) overnight at −20 °C. Tubes were centrifuged 25 min at 18516 *g* at 4 °C and washed once in ice cold ethanol 70%. The pellet was air dried and eluted using 60 μL of UltraPure water and stored at −80 °C until further use.

### Library Preparation and High-Throughput Illumina Sequencing

Sequencing libraries were prepared using the NEB Next Ultra II DNA Library Prep kit for Illumina (New England Biolabs, Ipswich, USA). Ten ng of IP DNA and 100 ng of input DNA were used (7 and 4 cycles of amplification by PCR were performed, respectively). Agencourt AMPure XP beads (Beckman Coulter, Brea, USA) were used for the 200 bp size selection of DNA fragments. Libraries concentrations were measured by Qubit and stored at −80 °C until sequencing. Single-end 50 bases sequencing was realized using an Illumina 4000 apparatus by the IGBMC GenomEast Platform (Illkirch, France).

### Bioinformatics Analysis

Sequencing quality was verified by a FastQC analysis (Babraham Bioinformatics). Duplicates were removed using SAMtools v1.0.1. Reads were aligned on the chicken genome Galgal5 [19] using Bowtie2 v2.2.6.2 (default options). Peak detection was realized using epic (version 0.1.23) [20], a re-implementation of SICER [21] (options: --fragment-size 50 --gaps-allowed 2 --false-discovery-rate-cutoff 0.05) on the chicken chromosomes of Galgal5 (scaffolds were excluded). H3K27me3 enrichment regions were determined for each sample individually. To assess the bias that may be linked to the heterogeneous number of unique reads between IP and input, we performed a random selection of 40 million of unique reads using a Perl script. Peak detection on this subset was realized using epic [20, 21], as described above. We determined the number of common peak using intersectBed (BEDTools v.2.25.0 [22]). Peaks were visualized with Integrative Genomics Viewer (Broad Institute) [23]. To produce the normalized BigWig files, a flagstat analysis (SAMtools) was first performed on the 40 million unique reads bam files to determine the number of mapped reads. The input data was then scaled to the corresponding H3K27me3 data using genomeCoverageBed (BEDTools; the scaling factor was determined by the ratio of the number of H3K27me3 mapped reads compared to the number of input mapped reads) and converted to bedGraph files. H3K27me3 bam files were also converted to bedGraph files using the same tool with no scaling factor. BigWig files were created using the bedGraphToBigWig tool and the normalized files [log2(H3K27me3/input)] were obtained using the bigwigCompare tool (default settings except for –pseudocount 0.1 and –bs 1).

### ChIP-qPCR

ChIP-qPCR primer sequences were designed using Primer3plus (Additional file 1: Table S1). Two microliters of ChIP DNA or 0.2 μL of input DNA were used with 5 μM of each primer and Takyon No ROX SYBR 2X MasterMix blue dTTP (Eurogentec, Liege, Belgium) following manufacturer’s instructions. Reactions were performed on a LightCycler® 480 Instrument (Roche Diagnostics, Basel, Switzerland) with the following program: denaturation 5 min at 95 °C, 50 amplification cycles (10 s at 95 °C, 15 s at 60 °C, 15 s at 72 °C), melting curve (5 s at 95 °C, 1 min at 65 °C, continuous at 95 °C) and cooling. Enrichments were determined with percent input method [100*2^ (adjusted input - Ct (IP)].

## Results

### Chromatin Preparation and Immunoprecipitation Performed Equivalently between X-ChIP and N-ChIP

In order to compare both fixed and native methods for preparing chromatin for ChIP-seq, we sampled in parallel 5 weeks-old male chicken muscle tissues (*Pectoralis major*) that were either cross-linked or snap frozen (unfixed). For cross-linked samples, we found that incubating muscle in the RBI relaxation buffer before fixation did improve tissue grinding by requiring less strength to grind despite this had no visible impact on chromatin shearing ([9] and data not shown). We determined the optimal cross-linking conditions by testing the following parameters: formaldehyde (FA) concentrations (1 and 2%), cross-linking duration (5 to 30 min) and sonication parameters (10 to 30 min with cycles 30 s ON / 30 s OFF) (Additional file 1: Fig. S1a). The condition that produced consistent shearing of DNA with an adequate fragment size of 200–600 bp was cross-linking with 1% FA for 5 min at room temperature followed by 15 min of sonication (Additional file 1: Fig. S1a lane 3). Similarly, we defined the Nuclease S7 Micrococcal nuclease (MNase) digestion conditions by adapting the duration of the enzyme incubation and its concentration (Additional file 1: Fig. S1b). Optimal mono-nucleosome digestion was achieved using 20 U of MNase incubated 15 min at 37 °C (Additional file 1: Fig. S1b lane 6). After chromatin preparation (Additional file 1: Fig. S1c-d), about twice more DNA was obtained for both X-ChIP input and IP fractions but the same amount of DNA was used to perform the library preparation (Additional file 1: Table S2).

### Fewer Regions of H3K27me3 Enrichment Were Identified by X-ChIP-Seq Compared to N-ChIP-Seq

**Fig. 1:**
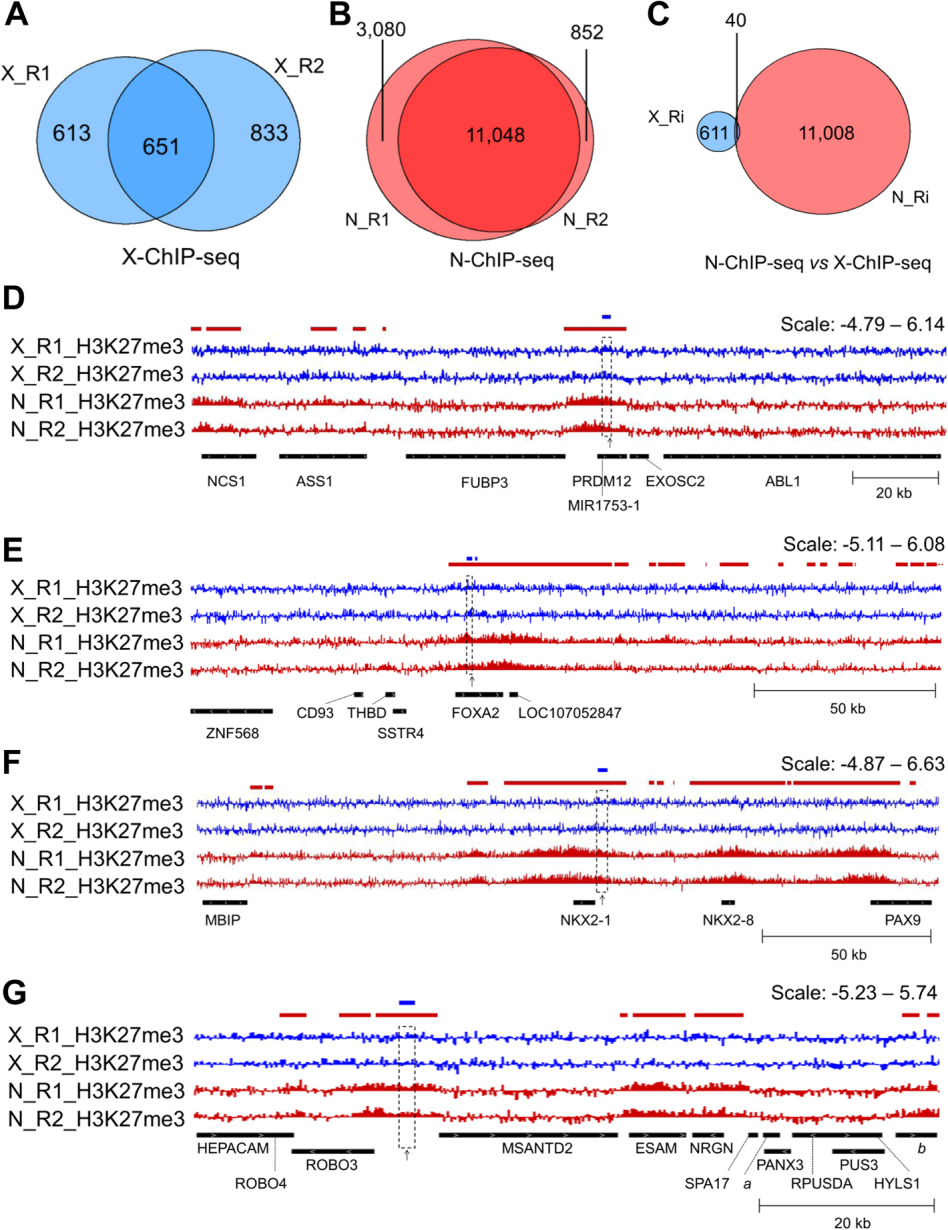
N-ChIP-seq and X-ChIP-seq results. **a**-**c** Venn diagrams representing the overlap determined by intersectBed between H3K27me3 broad peak regions detected by epic. **a** X-ChIP-seq replicates. **b** N-ChIP-seq replicates. **c** Intersection between X-ChIP-seq and N-ChIP-seq peaks (only common peaks for each condition were considered). **d**-**g** Visualization with IGV of H3K27me3 enrichment normalized to input [log2(IP/input)]. X-ChIP-seq muscle tracks are shown in blue and N-ChIP-seq muscle tracks in red. The colored boxes above the tracks represent broad peaks detected by epic in either X-ChIP-seq (blue) or N-ChIP muscle (red) replicates. The dashed boxes across the tracks represent the localization of a common peak (between methods). The black arrows under the tracks represent the position of qPCR primers used for Fig. 2A. **d** Common peak 1 (chr17: 6,422,600–6,424,599); (**e**) common peak 2 (chr3: 3,359,200–3,360,599); (**f**) Common peak 3 (chr5: 36,681,000–36,683,799); (**g**) common peak 4 (chr24:262,600–264,199), *a: LOC107055042, b: LOC101748751*

**Fig. 2:**
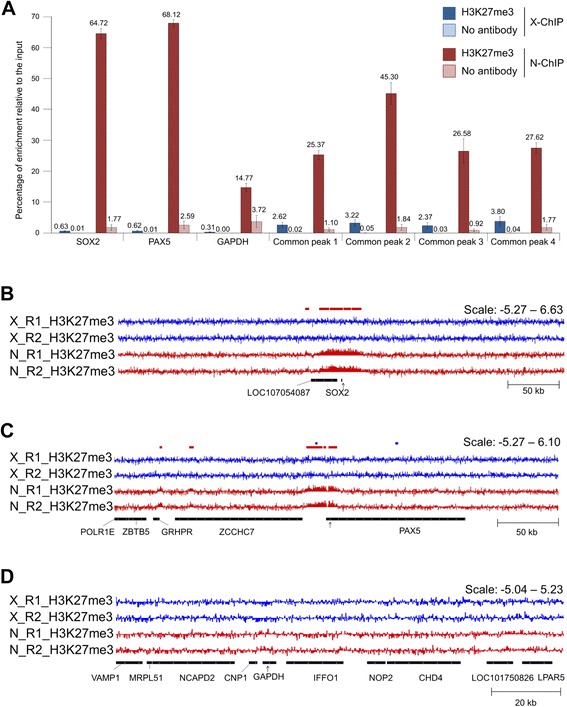
N-ChIP and X-ChIP results at control loci. **a** Enrichment of H3K27me3 and no antibody relative to the input measured by qPCR at three control gene loci: *SOX2* (chr9: 16,918,111–16,919,468), *PAX5* (chrZ: 81,789,479–81,896,738) and *GAPDH* (chr1: 76,950,864–76,956,805), and at the localization of common enrichment detected with epic (see Fig. 1 legend for peak description). H3K27me3 enrichments are represented in blue for X-ChIP and in red for N-ChIP, no-antibody enrichments are represented in light blue for X-ChIP and in light red for N-ChIP. Error bars: SEM. **b**-**d** Visualization with IGV of H3K27me3 enrichment normalized to input [log2(IP/input)]. X-ChIP-seq muscle tracks are shown in blue, N-ChIP muscle tracks are shown in red. The colored boxes above the tracks represent broad peaks detected by epic in either X-ChIP-seq (blue) or N-ChIP muscle (red) replicates. The black arrows under the tracks represent the position of qPCR primers at (**b**) *SOX2*, (**c**) *PAX5* and (**d**) *GAPDH*

Two biological replicates per ChIP method were sequenced using Illumina HiSeq technology (Table 1). The sequencing produced high-quality reads for both methods (q30 > 93%, Additional file 1: Fig. S2). We obtained 45.17 to 52.55 million of uniquely mapped reads for the input fractions and 69.58 to 106.36 million of uniquely mapped reads for H3K27me3 IP fractions. We next performed a broad peak detection analysis of H3K27me3 using epic that takes into account the enrichment context of a local window in determining its significance [20, 21]. On average ~2300 broad peaks were identified for X-ChIP-seq compared to ~15,000 broad peaks for N-ChIP-seq (Table 1). This represented ~6% of the genome enriched in H3K27me3 marks for N-ChIP-seq compared to only 0.75% for X-ChIP-seq. To be as comparable as possible for the analysis, we randomly selected 40 million of unique sequence for each sequencing fraction (IP and input) and individual which corresponds to the recommendations to study broad histone marks in Human and mice [5, 6, 24] (from now on data presented is derived from the 40 M sampling). While the 40 M sampling had a minor impact on the global number of peaks detected for N-ChIP-seq (85% of the initial number), it led to a lesser number of X-ChIP-seq peaks identified (59% of the initial number), corresponding to a genome coverage of 4.5% and 0.4% respectively (Table 1). The median peak length was globally higher for N-ChIP-seq compared to X-ChIP-seq (Additional file 1: Fig. S3). We analyzed the distribution of the peaks and found that half of them shared at least one base in common between both X-ChIP-seq replicates whereas 92.3% of the N-ChIP-seq peaks were common between replicates (Fig. 1a-b, Additional file 1: Fig. S4a-b). Peaks were preferentially located in regions around the TSS of genes as it was previously reported with a better defined signal around the TSS for N-ChIP-seq (Additional file 1: Fig. S4d) [16, 25]. Based on a previous transcriptome analysis using the same experimental design, we found that genes containing a peak displayed generally a low level of expression for both methods, in agreement with the repressive function of this mark (Additional file 1: Fig. S4d) [25]. We further compared the distribution of the 651 common X-ChIP-seq peaks and the 11,048 common N-ChIP-seq peaks and found that only 40 peaks intersected, which is less than 1% of the total number of identified peaks (Fig. 1c). This surprising finding is consistent with the peak distribution that appeared different between both methods (Additional file 1: Fig. S4c). Genomic regions of enrichment in H3K27me3 were visualized using IGV [23]. While N-ChIP-seq H3K27me3 broad peak signal could be clearly identified, X-ChIP-seq signal appeared more discreetly at common peak positions (Fig. 1d-g and Fig. 2c). These results were confirmed using another peak caller (MACS2 [26]): only 38 and 32 non-overlapping regions of enrichment were identified for both X-ChIP-seq replicates compared to 17,113 and 17,591 for N-ChIP-seq replicates with 65% of overlap (Additional file 1: Table S3 and Fig. S5).

**Table 1:** Sequencing results of cross-linked and native ChIP-seq on muscle samples. Two biological replicates were sequenced for each chromatin immunoprecipitation method (X-ChIP and N-ChIP). Reads were mapped against the chicken genome Galgal5, and peaks were detected using epic. When indicated (40 M), analyses were performed on 40 million of unique reads per sample that were randomly selected for both H3K27me3 and input fractions. Genome coverage is expressed in Megabases

Method	X-ChIP				N-ChIP			
Sample	X_R1		X_R2		N_R1		N_R2	
Fraction	Input	H3K27me3	Input	H3K27me3	Input	H3K27me3	Input	H3K27me3
Total number of reads (millions)	57.37	119.58	60.29	123.37	57.98	92.93	58.94	128.56
Total number of uniquely mapped reads (millions)	51.16	106.36	52.55	104.29	45.17	69.58	46.51	92
Total peak number	2127		2519		16,446		13,949	
Total genome coverage (Mb)	7.87		9.75		73.52		71.14	
40 M peak number	1264		1484		14,128		11,900	
40 M genome coverage (Mb)	4.76		5.21		60.51		51.21

### The Lower X-ChIP-Seq Enrichment was Confirmed by ChIP-qPCR

H3K27me3 enrichment was verified by ChIP-qPCR on three biological replicates for which chromatin was prepared both in native and under cross-linked conditions. We investigated seven regions of interest. Three regions were selected based on the literature, two of them known to be enriched for the H3K27me3 mark in myotubes and myoblasts (*SOX2* and *PAX5* [25]) and one known to display low levels of enrichment (*GAPDH* [27]). Four other regions were selected as common regions of enrichment identified in the present ChIP-seq experiment (Fig. 2). The epic analysis was able to detect enrichments at both *SOX2* and *PAX5* loci for N-ChIP-seq but failed to detect a peak at *SOX2* locus for X-ChIP-seq (as shown by red and blue bars in Fig. 2b-c). As expected, the H3K27me3 enrichment relative to the input was observed at all loci tested with lower levels at the *GAPDH* locus (Fig. 2a). In addition, the percentage of H3K27me3 enrichment relative to the input was significantly lower for X-ChIP-seq than for N-ChIP-seq at the different loci tested, notably at the *SOX2* (0.62% and 64.72% in average respectively) and *PAX5* loci (0.62% and 68.95% in average respectively; Fig. 2b-c).

### H3K27me3 X-ChIP-Seq was Suitable to Detect Broad Peaks in Chicken Hypothalamus

Despite chromatin preparation seemed to perform normally for cross-linked muscle samples (Additional file 1: Fig. S1c), we tested our fixed chromatin preparation protocol on another tissue that was not previously reported to be problematic for X-ChIP-seq. To that end we performed fixation and chromatin preparation on two hypothalamus samples from 5 weeks-old male chickens (Additional file 1: Supplemental methods). The chromatin was fragmented similarly to the muscle chromatin (Additional file 1: Fig. S6a) and Illumina sequencing was performed in the same conditions except for a larger number of amplification cycles used during the library creation process (10 instead of 7) due to the lower amount of chromatin extracted from the 30 mg samples. About 13,000 broad peaks were identified using epic, representing about 59% of the genome covered in H3K27me3 (Additional file 1: Table S4). In addition, H3K27me3 enrichment levels could be clearly visualized at the *SOX2* and *PAX5* control loci (Additional file 1: Fig. S6b-d), similarly to what was observed for the N-ChIP-seq muscle signals. Altogether, this suggests that our X-ChIP-seq protocol performed adequately and that the issues reported above may be specifically related to the muscle tissue used.

## Discussion

Chromatin fixation and fragmentation by sonication or MNase digestion are key steps for a successful ChIP experiment. In this study, we sought to compare both chromatin preparation strategies to perform ChIP-seq from chicken skeletal muscle tissue. We determined first the optimal conditions for chromatin fixation and shearing (X-ChIP) or enzymatic digestion (N-ChIP). For X-ChIP, we found that muscle chromatin shearing was optimal when the tissue was fixed for 5 min in 1% FA and chromatin was sonicated for 15 min, resulting in DNA fragments ranging from 100 to 600 bp as described previously [6, 28]. Therefore, despite reports suggesting that cross-linking reagents may not be efficient to properly fix the chromatin in muscle tissue [8], we were able to produce chromatin from skeletal muscle that was appropriately sheared [6, 28]. For N-ChIP preparation, 15 min of MNase digestion at a concentration of 20 U produced mono-nucleosomes as recommended [29]. We used comparable amount of chromatin for the IP, about 6 μg for X-ChIP and 5 μg for N-ChIP, and the same batch of anti-H3K27me3 antibody was used [6]. X-ChIP immunoprecipitation yielded about twice as much chromatin as N-ChIP but the same observation was made for input DNA recovery, suggesting that this difference may be due to the purification method that differed between both approaches (as X-ChIP included a cross-linking reversal step) [17, 18]. Therefore, until this point, both ChIP methods seemed to perform adequately and in a relatively similar manner.

The same amount of chromatin was used for the library preparation and sequencing. Four libraries (two per method) were sequenced for at least 40 million unique reads following ENCODE ChIP-seq guidelines for broad source signals [5, 6]. As the histone mark H3K27me3 is known to be distributed in peaks spanning across broad regions, we analyzed the data using epic, a ChIP-seq caller based on the SICER algorithm [20, 21]. Our analysis showed that H3K27me3 genome coverage was 10-fold higher for N-ChIP-seq compared to X-ChIP-seq (Table 1). Less than 1% of the genome was covered in peaks for X-ChIP-seq. To make sure that this result was not due to the different number of reads between all input and IP samples, we performed the analysis on 40 million of unique reads for each input and IP sample, following ENCODE recommendations [5]. However, this had no major impact on the number of peaks identified (Table 1). Strikingly, N-ChIP-seq peak regions were highly consistent between replicates (> 90% of peaks were common for N-R2 compared to N-R1) while only about half of the peaks detected in X-ChIP-seq data were common to both replicates (Fig. 1a-b). While we cannot exclude that this may be due to a difference in the biological replicates used in this study (despite same genotype and rearing condition were used), we would rather speculate that this is likely due to the fact that peaks are better defined in the N-ChIP-seq analysis, as seen on the IGV genome browser for common peaks regions (Fig. 1 d-g). These results would therefore suggest that the peak detection is more robust for N-ChIP-seq data. Differences were even more dramatic when analyzed with the peak caller MACS2 as only about 30 peaks were identified for X-ChIP-seq compared to about 17,000 peaks identified for N-ChIP-seq. It is worth noting that epic performed better in terms of peak discovery compared to MACS2 for X-ChIP-seq, which is in agreement with the fact that epic may be better suited to identify broad source signals such as H3K27me3 than the most-widely used MACS2 tool.

Despite ENCODE ChIP-seq guidelines recommended at least two biological replicates [6], we acknowledge that only two replicates is not optimal for performing a proper, unbiased detection of peaks. However, this study showed that epic led to a remarkably reproducible identification of peaks between replicates for N-ChIP-seq. In addition, we verified H3K27me3 enrichments for 3 biological replicates by ChIP-qPCR at control loci (*PAX5* and *SOX2*) and peaks from the ChIP-seq analysis (Fig. 2a). ChIP-qPCR results were in agreement with the lower peak signal observed for the X-ChIP-seq compared to the N-ChIP-seq, supporting the fact that native preparation of chromatin led to a stronger H3K27me3 enrichment signal at loci of interest after immunoprecipitation.

Surprisingly, we found that less than only 1% of the X-ChIP-seq peaks intersected with those from the N-ChIP-seq (Fig. 1c). This difference is in agreement with the non-overlapping peak distribution reported by the ChIP-seeker analysis, in particular at the Z chromosome (Additional file 1: Fig. S4c). These observations suggest that the majority of the peaks identified by both methods were distinct. It was reported previously that MNase digestion may lead to a selective digestion of particular chromatin domains during preparation, and that the fixation may also induce signal artifacts caused by the crosslinking with other genomic regions [1, 29]. However, none of the studies reported such a discrepancy in the peak distribution. This suggests that one or several factors in the *P. major* cellular environment may affect the chromatin conformation, accessibility and/or shearing in a significant manner. Nonetheless, in addition to a much larger number of peaks identified, N-ChIP-seq signal was consistent with the H3K27me3 signal previously reported for other tissues and species, as illustrated by the *SOX2* and *PAX5* control regions profiles of enrichment. Therefore, while some peaks may be missed by N-ChIP-seq in chicken breast muscle for unknown reasons that would require further studies, the native preparation of chromatin appeared to be the most suitable to detect a majority of the expected peaks.

Given that X-ChIP-seq protocol seemed to be less efficient than N-ChIP-seq protocol on *P. major* tissue, we verified that the X-ChIP-seq protocol functioned properly on tissues for which nuclei were easily accessible. To that end we used chicken brain tissue (hypothalamus). Cross-linking and chromatin fragmentation of hypothalamus gave similar results in terms of chromatin shearing. The H3K27me3 X-ChIP-seq signal from hypothalamus was clearly distinct from the input fraction signal at *PAX5* and *SOX2* control loci contrary to the muscle X-ChIP-seq signal (Additional File 1: Fig. S6). The epic analysis produced a number of peaks similar to the one observed for N-ChIP-seq from muscle. Moreover, the number of enriched regions in hypothalamus X-ChIP-seq and muscle N-ChIP-seq were comparable to what Luo and co-workers have reported in chicken spleen by N-ChIP-seq for this mark [30]. These results suggest that our method succeeded in identifying regions of enrichment using cross-linked chromatin from brain, despite much less starting material was used.

Two studies successfully used X-ChIP-seq on muscle tissues to study narrow histone marks and transcription factors [14, 15]. However, detecting narrow histone modifications such as H3K4me3 is often easier than broad marks detection due to their sharp distribution on the genome, as illustrated by the lower sequencing depth requirement for studying these marks [5, 6, 24]. Altogether, these results support the hypothesis that the relatively low performance of X-ChIP-seq from chicken *P. major* muscle is likely to be due to the poor compatibility of the chromatin preparation method with this particular tissue.

## Conclusions

We performed in parallel two ChIP-seq analyses of the H3K27me3 mark from native and fixed chromatin extracted from chicken skeletal muscle tissue (*P. major*). Our analysis showed that in skeletal muscle X-ChIP-seq was much less efficient as 10-times less regions of enrichment (peaks) were detected compared to N-ChIP-seq on the same tissues. We therefore recommend performing N-ChIP-seq to characterize HPTM at a whole genome scale in chicken skeletal tissue samples, possibly extending to other species including mammals.

## Additional file

Additional file 1: Fig. S1. Setup of chromatin preparation conditions.** Table S1.** ChIP-qPCR primers. **Table S2.** Quantity of chromatin and DNA used throughout the protocol. **Fig. S2.** Quality assessment of the sequencing reads. **Fig. S3.** Boxplot representing the distribution of the 40 M peaks lengths. **Fig. S4.** X-ChIP-seq and N-ChIP-seq peaks analysis. **Table S3.** Broad peak detection analysis by MACS2 for both H3K27me3 X-ChIP-seq and N-ChIP-seq experiments. **Fig. S5.** Comparison of H3K27me3-enriched peak regions detected by MACS2 and between MACS2 and epic. Supplemental methods X-ChIP-seq protocol for hypothalamus tissue samples. **Table S4.** Sequencing results of hypothalamus X-ChIP-seq analyzed using epic. **Fig. S6.** Fragmentation assessment of chromatin before immunoprecipitation for hypothalamus samples and X-ChIP results at three control loci. (PDF 1487 kb)

## Abbreviations

CDB: ChIP dilution buffer
ChIP-qPCR: Chromatin immunoprecipitation followed by qPCR
ChIP-seq: Chromatin immunoprecipitation followed by high throughput sequencing
FA: Formaldehyde
H3K*me3: Tri-methylation of lysine * on the histone H3 (* can be 4, 27 or 36 in this article)
HPTM: Histone post-translational modifications
IP: Immunoprecipitation
MNase: Micrococcal nuclease
N-ChIP: Chromatin immunoprecipitation on native chromatin
PBS: Phosphate buffered saline
PBS-C: phosphate buffered saline supplemented with Complete™ protease inhibitors
qPCR: Quantitative polymerase chain reaction
RBI: Relaxation buffer
SDS: Sodium dodecyl sulphate
X-ChIP: Chromatin immunoprecipitation on cross-linked chromatin
