# Supplementary material for: An Assessment of Fixed and Native Chromatin Preparation Methods to Study Histone Post-Translational Modifications at a Whole Genome Scale in Skeletal Muscle Tissue
Source: Biol Proced Online. 2017 Aug 29;19:10. doi: 10.1186/s12575-017-0059-0 (PMC5576305; doi:10.1186/s12575-017-0059-0)
Supplement: Additional file 1: Fig. S1. — Setup of chromatin preparation conditions. Table S1. ChIP-qPCR primers. Table S2. Quantity of chromatin and DNA used throughout the protocol. Fig. S2. Quality assessment of the sequencing reads. Fig. S3. Boxplot representing the distribution of the 40 M peaks lengths. Fig. S4. X-ChIP-seq and N-ChIP-seq peaks analysis. Table S3. Broad peak detection analysis by MACS2 for both H3K27me3 X-ChIP-seq and N-ChIP-seq experiments. Fig. S5. Comparison of H3K27me3-enriched peak regions detected by MACS2 and between MACS2 and epic. Supplemental methods X-ChIP-seq protocol for hypothalamus tissue samples. Table S4. Sequencing results of hypothalamus X-ChIP-seq analyzed using epic. Fig. S6. Fragmentation assessment of chromatin before immunoprecipitation for hypothalamus samples and X-ChIP results at three control loci. (PDF 1487 kb) [file 12575_2017_59_MOESM1_ESM.pdf]

**Additional file 1**

**Figure S1: Setup of chromatin preparation conditions.** (A) Gel electrophoresis (1% agarose stained with GelRed, Biotium, Fremont, USA) of cross-linked and sonicated chromatin. Cross-linking was reversed before migration. Lane 1: GeneRuler 1 kb DNA Ladder, ref. SM0311, Thermo-Scientific, Waltham, USA. Lanes 2 to 5: FA 1 % for 5 min followed by respectively 2 x 5, 3 x 5, 4 x 5 or 6 x 5 min of sonication. Lanes 6 to 8: FA 1 % for 15 min followed by respectively 2 x 5, 4 x 5 or 6 x 5 min of sonication. Lanes 9 to 11: FA 2 % for 30 min followed by respectively 2 x 5, 4 x 5, 6 x 5 min of sonication. (B) Gel electrophoresis (1% agarose stained with GelRed) of Mnase digested chromatin. Lane 1: GeneRuler 1 kb DNA Ladder. Lane 2: 10 U Mnase for 7 min. Lane 3: 10 U Mnase for 10 min. Lane 4: 20 U Mnase for 10 min. Lane 5: 10 U Mnase for 15 min. Lane 6: 20 U Mnase for 15 min. Lane 7: GeneRuler 50 bp DNA Ladder, ref. SM0371, Thermo-Scientific, Waltham, USA. (C) Gel migration (1% agarose stained with GelRed) of muscle X-ChIP chromatin samples used for the sequencing. Lane 1: GeneRuler 50 bp DNA Ladder. Lane 2: Replicate 1 (X_R1). Lane 3: Replicate 2 (X_R2). Lane 4: GeneRuler 1 kb DNA Ladder. (D) Gel migration (1% agarose stained with GelRed) of muscle N-ChIP chromatin samples used for the sequencing. Lane 1: Replicate 1 (N_R1). Lane 2: Replicate 2 (N_R2). Lane 3: 100 bp DNA marker ref. 523002, Ready-to-use DNA Markers: 100 bp, Dominque Dutscher, Brumath, France.

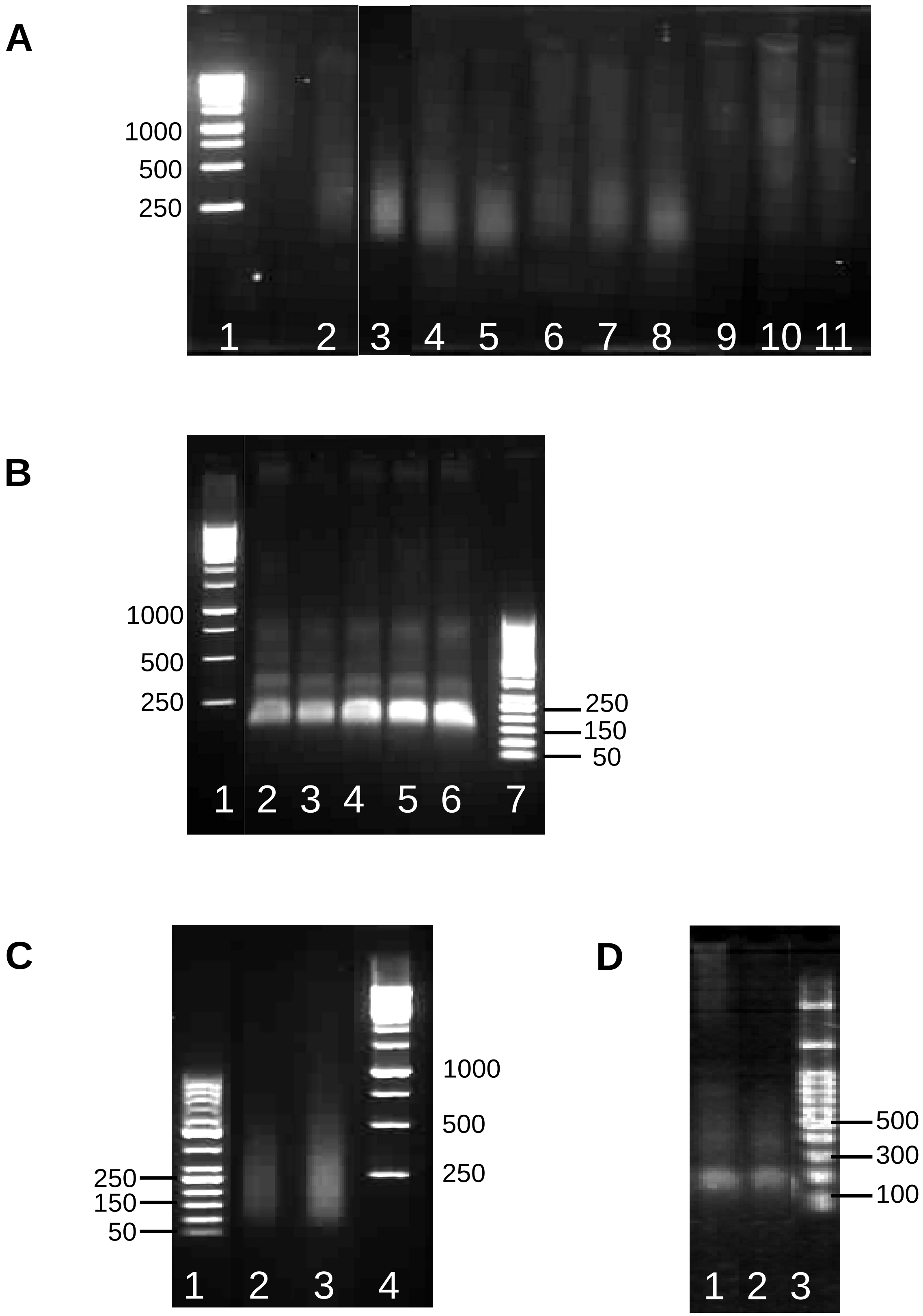


David et al.: Additional File 1_Figure S1

**Table S1: ChIP-qPCR primers**

| Primer name | Forward primer | Reverse primer | Product size (bp) |
|---|---|---|---|
| PAX5 | 5’-GCATTTGAGTTGTCAGTCTATTC-3’ | 5’-GTGCTACCCGTCCGAATTAC-3’ | 158 |
| SOX2 | 5’-AACGGATCGCCTACCTACAG-3’ | 5’-GAATGAGACGAGGAGGTGACTAC-3’ | 193 |
| GAPDH | 5’-AGACAGGAACAAGGCGTAGTG-3’ | 5’-CCTTGAAGTGTCCGTGTGTAG-3’ | 131 |
| Common peak 1 | 5’-GGTGGGTGGGATGGTTATC-3’ | 5’-GCTGTCTGTGTACAGGGAATTAAAC-3’ | 103 |
| Common peak 2 | 5’-AGCCTGCGGGTAGGAAATAG-3’ | 5’-GCAGAACAAGACGCCTACAG-3’ | 96 |
| Common peak 3 | 5’- TCCAGGTGTTCCCTTTGAGTAG-3’ | 5’-GGTCTCTTCCAATCTGGGATAC-3’ | 91 |
| Common peak 4 | 5’-TCCCATTTCCAGCTCTTTAATAC-3’ | 5’-CTTCGCACATGAGCCACTAC-3’ | 162 |

**Table S2: Quantity of chromatin and DNA used throughout the protocol.** Quantification of chromatin kept for input or used for immunoprecipitation was estimated by spectrophotometry (NanoDrop ND-1000 Spectrophotometer). For all subsequent steps, DNA concentrations were determined by Qubit Fluorometric Quantitation (Qubit dsDNA Assay Kit, Thermo Scientific, Waltham, USA).

| Protocol | X-ChIP | | | | N-ChIP | | | |
|---|---|---|---|---|---|---|---|---|
| Sample | X_R1 | | X_R2 | | N_R1 | | N_R2 | |
| Fraction | input | H3K27me3 | input | H3K27me3 | input | H3K27me3 | input | H3K27me3 |
| Chromatin kept for input or used for IP (µg) | 1.8 | 6 | 1.8 | 6.4 | 2.5 | 5 | 2.5 | 5 |
| DNA obtained after reverse-crosslink and purification or IP (ng) | 604.42 | 59.19 | 746.68 | 96.27 | 301.86 | 26.1 | 477.31 | 41.83 |
| DNA used for library preparation (ng) | 100.17 | 10.14 | 100.25 | 10.18 | 100.62 | 10.09 | 100.77 | 10.23 |
| DNA obtained from library preparation (ng) | 129.74 | 84.27 | 126.60 | 65.43 | 177.48 | 129.30 | 143.85 | 174.37 |

**Figure S2: Quality assessment of the sequencing reads.** Quality was determined by the FastQC q30 value (Babraham Bioinformatics, version 0.11.2, arguments --nogroup –casava). q30 is the percentage of bases above a Qscore of 30. (A) H3K27me3 X_R1 reads from muscle, q30: 94.05 %; (B) Input X_R1 reads from muscle, q30: 93.98 %; (C) H3K27me3 X_R2 reads from muscle, q30: 93.98 %; (D) Input X_R2 reads from muscle, q30: 94.16 %; (E) H3K27me3 N_R1 reads from muscle, q30: 97.21 %; (F) Input N_R1 reads from muscle, q30: 97.31 %; (G) H3K27me3 N_R2 reads from muscle, q30: 97.15 %; (H) Input N_R2 reads from muscle, q30: 97.22 %; (I) H3K27me3 HT_X_R1 reads, q30: 97.28 %; (J) Input HT_X_R1 reads, q30: 97.39 %; (K) H3K27me3 HT_X_R2 reads, q30: 96.89 %; (L) Input HT_X_R2 reads, q30: 96.84 %.

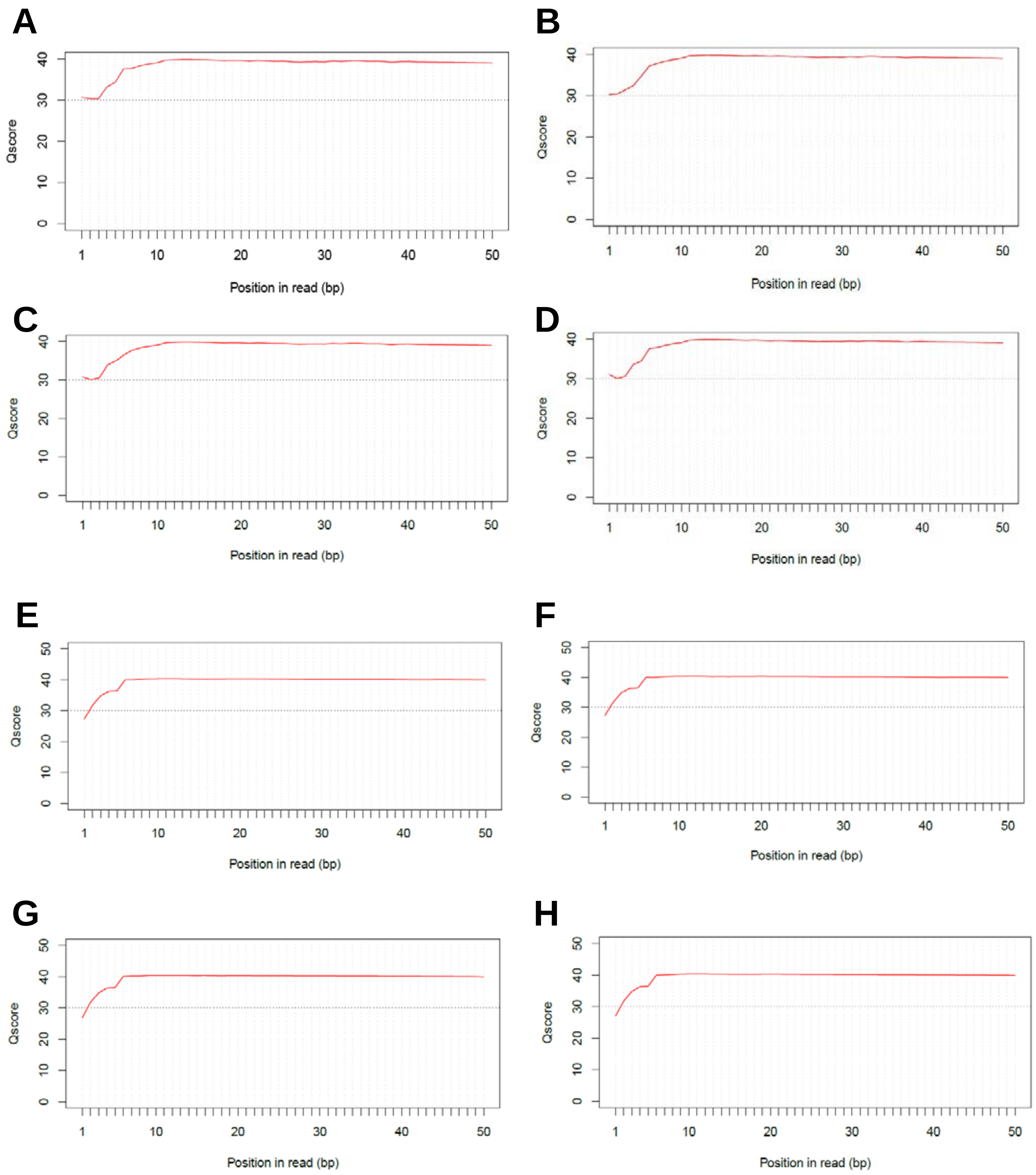


David et al.: Additional File 4_Figure S2

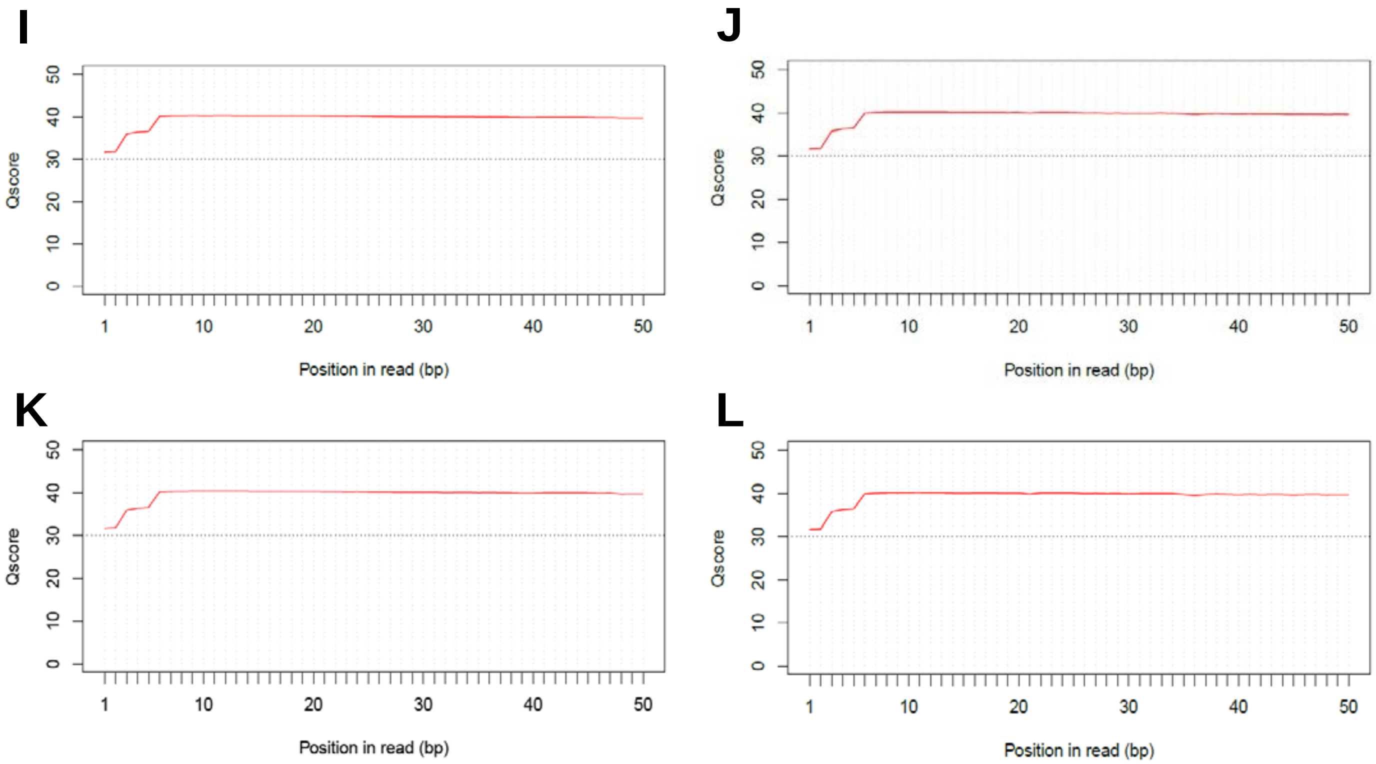


David et al.: Additional File 4_Figure S2

**Figure S3: Boxplot representing the distribution of the 40M peaks lengths.** Boxplots of peak width (A) with or (B) without outliers (using the argument outline=F) were determined using the R boxplot function on 40M datasets. Common peaks between X-ChIP-seq replicates are shown in blue (median width: 2000 bp), N-ChIP-seq common peaks in red (median width: 2600 bp).

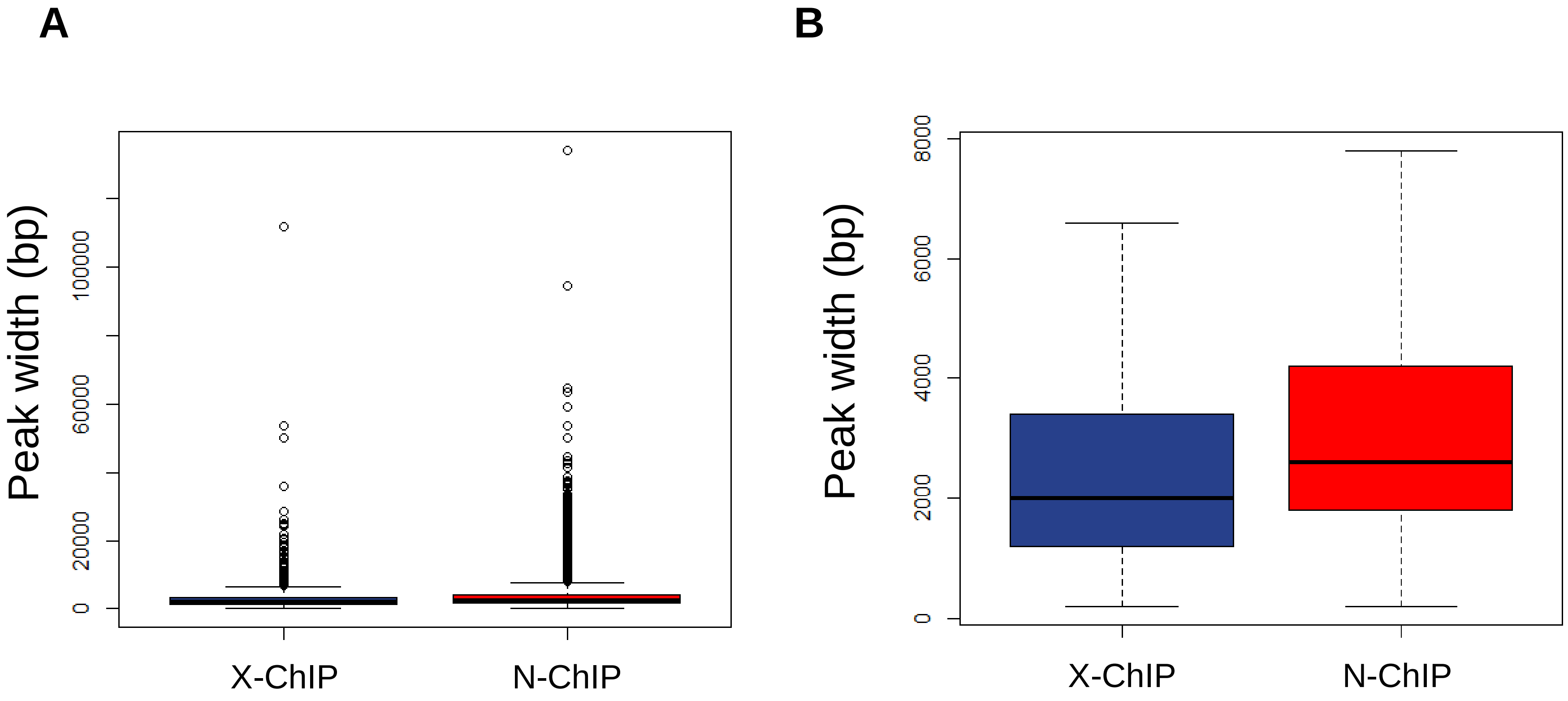


David et al.: Additional File 5 Figure S3

**Figure S4: X-ChIP-seq and N-ChIP-seq peaks analysis**. (A-C) Covplots were calculated with ChIPseeker on 40M datasets (Yu G, Wang L and He Q, 2015). (A) Covplot of X-ChIP-seq common peaks; (B) Covplot of N-ChIP-seq common peaks; (C) Covplot of Merged covplot of both X-ChIP-seq peaks (blue) and N-ChIP-seq peaks (red); (D) Profile of common peak data around the TSS (+/- 10 Kb of the TSS) obtained using the plotAvgProf command of ChIPseeker. X_Ri: common X-ChIP-seq peaks, N_Ri: common N-ChIP-seq peaks; (E) Correlation analysis of H3K27me3 peak localization with relative expression value. Transcriptome data from Loyau T *et al.* (2016) was used. Expression values were computed from microarray data (8 x 60 K) corresponding to male control animals of the same age (35d), line (Cobb500) and tissue (*P. major*) for all probes. Probe sequences were aligned against the Galgal5 genome (NCBI) using blastn (default parameters) and the probes displaying 100% of identity were kept, corresponding to 13965 unique genes with expression values. The intersection of expression values and peaks was performed in R (R Development Core Team, 2008) using Rstudio (RStudio Team, 2015) based on the following criteria: presence of a peak within the gene coordinates (5'UTR-3'UTR). The CRAN package ggplot2 was used to generate the density plot (left) and the boxplot (right) of mean expression values for all genes and the genes that contained a peak from the N-ChIP-seq (N-Ri) or X-ChIP-seq (X-Ri) analysis (only common peaks between replicates were used).

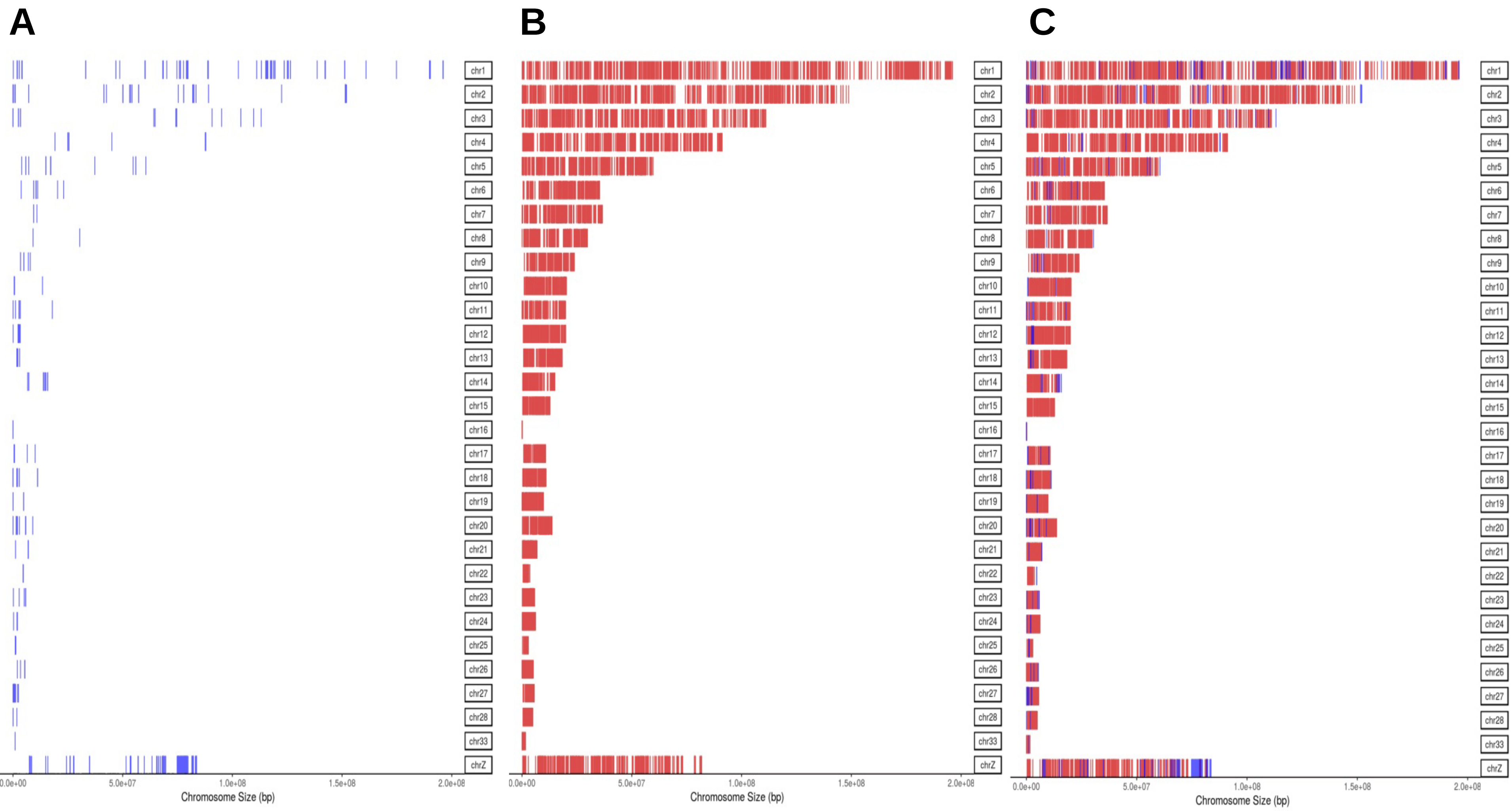


David et al.: Additional File 6 Figure S4

**D**

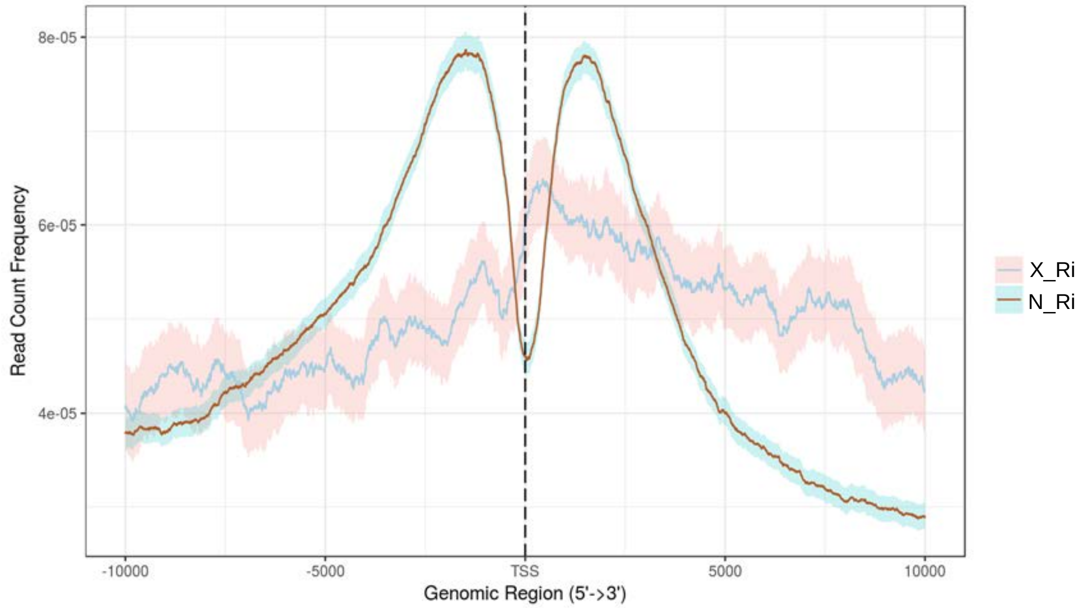


**E**

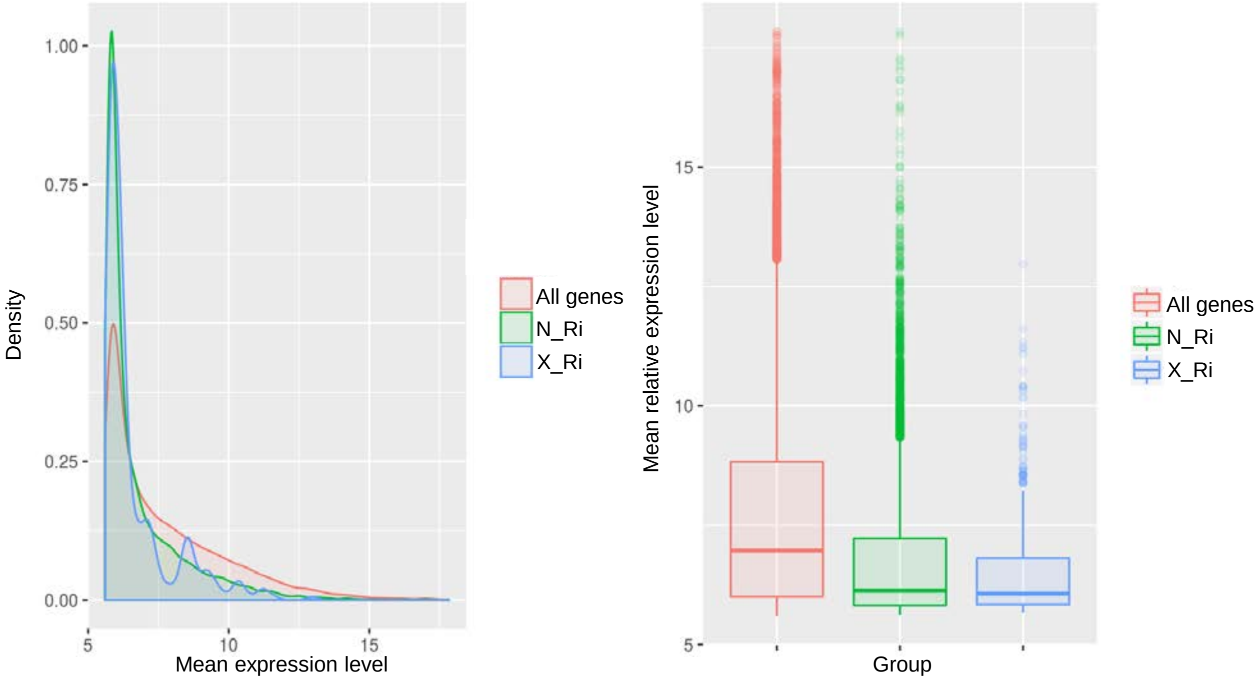


David et al.: Additional File 6 Figure S4

**Table S3: Broad peak detection analysis by MACS2 for both H3K27me3 X-ChIP-seq and N-ChIP-seq experiments**. 40 million of unique reads per sample were randomly selected among the uniquely mapped reads for each replicate and broad peak detection was performed with the peak caller MACS2 (MACS2_callpeak 2.1.0.20140616, bandwidth 300, q-value 0.05, build model OFF, broad peaks ON).

| Method | X-ChIP | | N-ChIP | |
|---|---|---|---|---|
| Sample | X_R1 | X_R 2 | N_R 1 | N_R 2 |
| Peak number | 38 | 32 | 17113 | 17591 |
| Genome coverage (Mb) | 0.0186 | 0.0154 | 36.60 | 35.36 |

**Figure S5: Comparison of H3K27me3-enriched peak regions detected by MACS2 and between MACS2 and epic**. (A) Venn diagram representing the intersection between broad peak regions detected by MACS2 for both X-ChIP-seq replicates. No common regions were found. (B) Venn diagram representing the overlap between broad peak regions detected by MACS2 for N-ChIP-seq replicates. (C) Venn diagram representing the intersection between broad peak regions common to both N-ChIP-seq replicates detected by epic and MACS2.

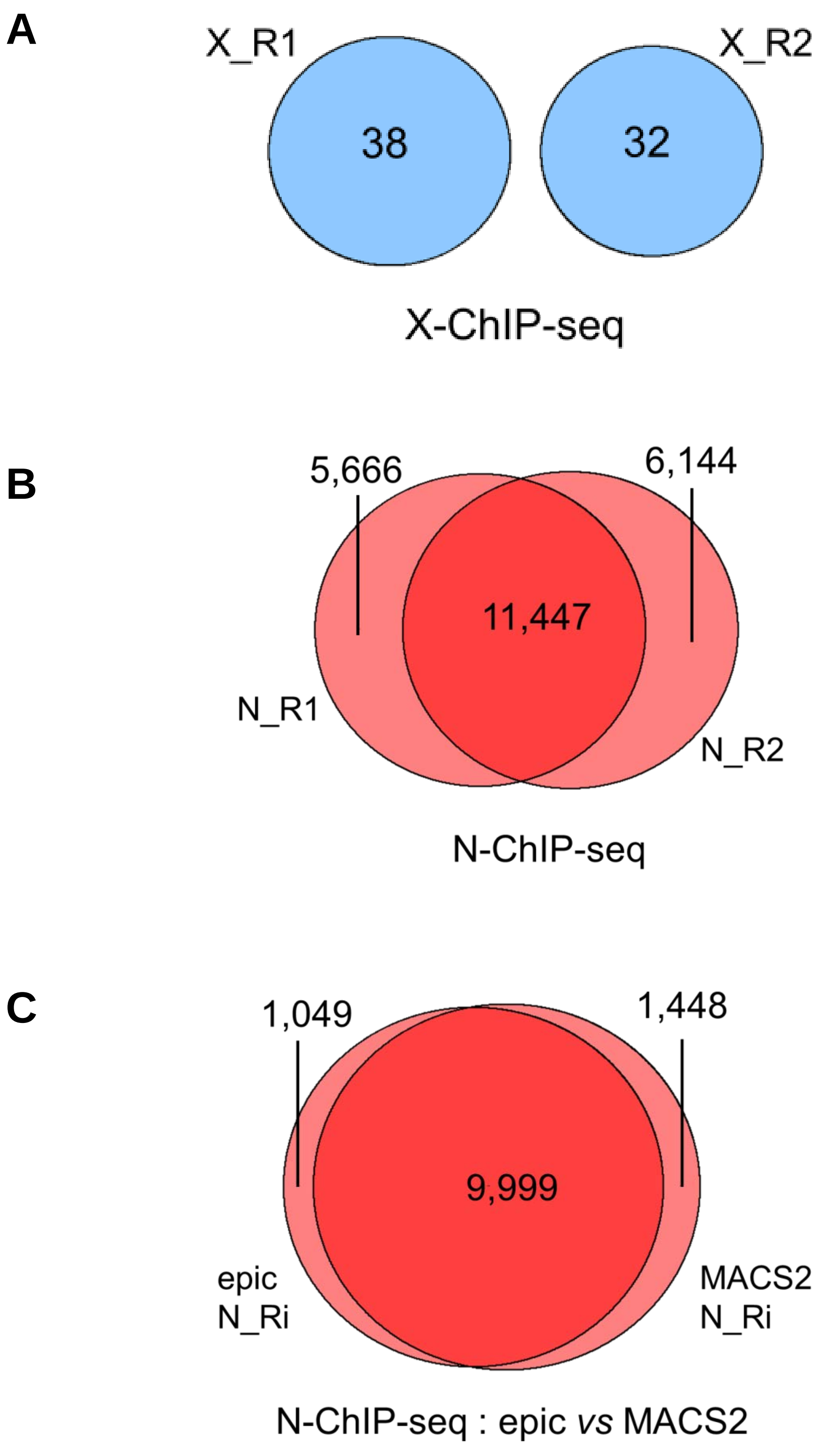


David et al.: Additional File 7_Figure S5

**Supplemental methods: X-ChIP-seq protocol for hypothalamus tissue samples**

Hypothalamus tissues were snap frozen at sampling. Samples were ground in a mortar with a pestle cooled with liquid nitrogen then resuspended in 900 µL of ice-cold PBS-C and transferred to 1.5 mL TPX tubes. 100 µL of crosslink buffer was added (100 mM NaCl, 1 mM EDTA pH 8, 0.5 mM EGTA pH 8, 50 mM HEPES pH 8, 1 % formaldehyde) and tubes were incubated 5 min at room temperature under shaking. The reaction was stopped by adding glycine to a final concentration of 0.125 M for 5 min at room temperature under shaking. Pellets were washed once in PBS-C and then incubated 10 min in 300 µL of SDS lysis buffer (50 mM Tris-HCl pH 8, 20 mM EDTA pH 8, 1 % SDS, 1 X Complete™ protease inhibitors). Samples were vortexed and incubated 10 min on ice, then sonicated 4 x 5 min using the Bioruptor (high – 30 s ON / 30 s OFF). Samples were centrifuged 5 min at 10600 *g* and the supernatant containing the chromatin was collected and used fresh or stored at -80 °C.

Chromatin quantification, quality assessment, immunoprecipitation and Illumina sequencing were performed as described for the muscle (2.45 ng for HT_X_R1 and 1.1 ng for HT_X_R2 of IP DNA and 100 ng of input DNA were used to prepare the sequencing libraries).

**Table S4: Sequencing results of hypothalamus X-ChIP-seq analyzed using epic.** Two biological replicates were sequenced (HT_X_R1 and HT_X_R2). Reads were mapped against the Galgal5 chicken genome.

| Method | X-ChIP | | | |
|---|---|---|---|---|
| Sample | HT_X_R1 | | HT_X_R2 | |
| Fraction | Input | H3K27me3 | Input | H3K27me3 |
| Total number of uniquely mapped reads (M) | 42.44 | 94.64 | 29.23 | 112.77 |
| Total peak number | 13133 | | 12531 | |
| Total genome coverage (Mb) | 59.70 | | 59 | |
| Number of common peaks between replicates | 11308 | | | |

**Figure S6: Fragmentation assessment of chromatin before immunoprecipitation for hypothalamus samples and X-ChIP results at three control loci.** (A) 1% agarose stained with GelRed was used for the chromatin migration. Lane 1: GeneRuler 50 bp DNA Ladder, ref. SM0371, Thermo-Scientific, Waltham, USA. Lane 2: Replicate 1 (HT_X_R1). Lane 3: Replicate 2 (HT_X_R2). Lane 4: GeneRuler 1kb DNA Ladder, ref. SM0311, Thermo-Scientific, Waltham, USA. (B-D) IGV visualization of H3K27me3 40M unique reads signal in the hypothalamus tissue at control loci. H3K27me3 enrichments were normalized to the input [log2(IP/input)]. The green boxes represent broad peaks detected by epic. (B) *SOX2* (chr9: 16918111-16919468); (C) *PAX5* (chrZ: 81789479-81896738); (D) GAPDH (chr1: 76950864-76956805) loci.

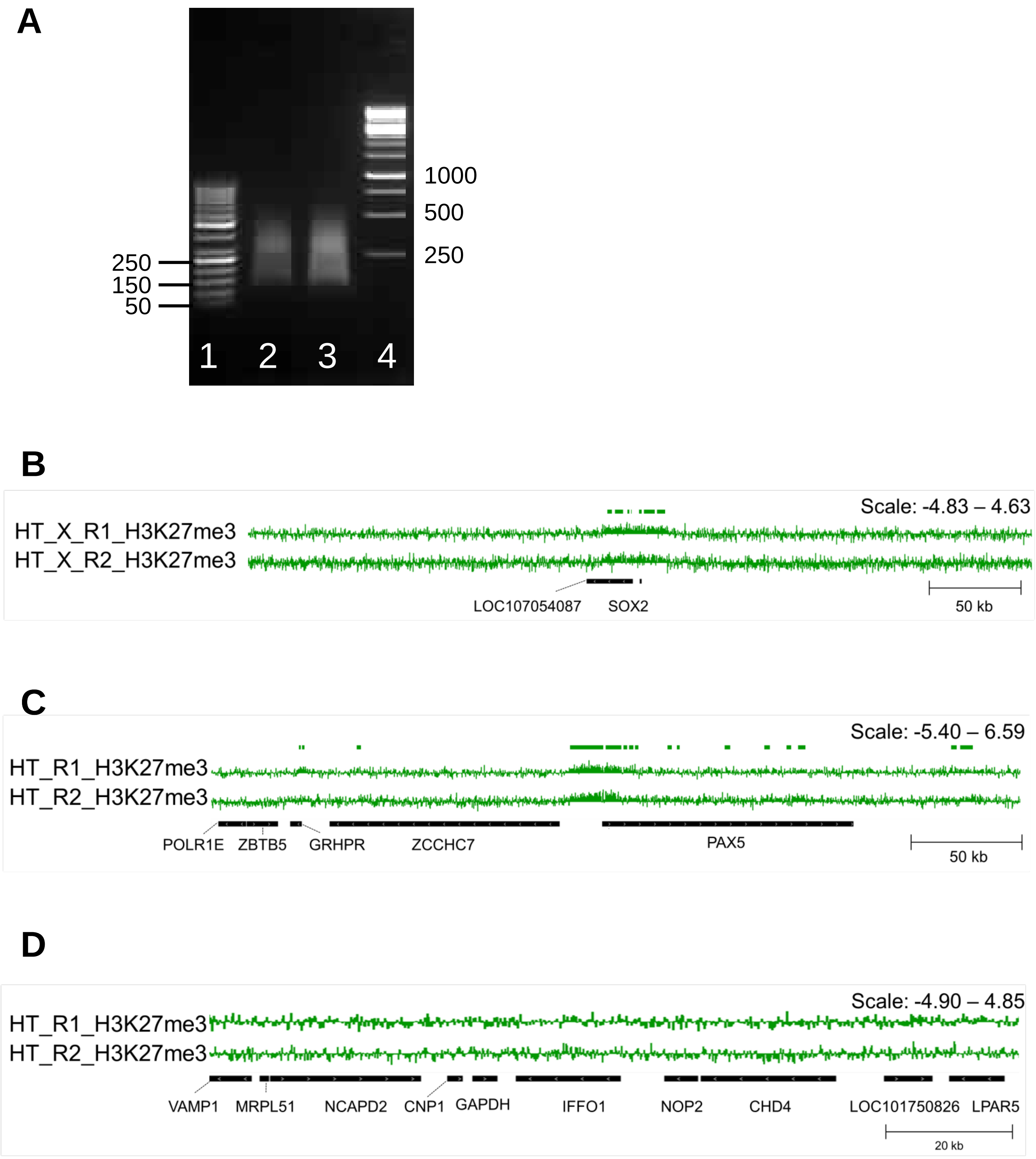


David et al.: Additional File 8_Figure S6
